# Robotic sigmoid colectomy with intracorporeal anastomosis: IMV first approach—a video vignette

**DOI:** 10.1007/s10151-025-03249-6

**Published:** 2026-03-27

**Authors:** Valentin Butnari, Ahmer Mansuri, Matthew Hanson, Richard Boulton, Joseph Huang, Nirooshun Rajendran, Sandeep Kaul

**Affiliations:** 1 Department of Surgery, Barking, Havering and Redbridge University Hospitals NHS Trust, London, UK; 2 The Centre for Neuroscience, Surgery and Trauma, Blizard Institute, Faculty of Medicine and Dentistry, Queen Mary University of London, National Bowel Research Centre, London, UK

**Keywords:** Colon cancer, Colorectal surgery, Robotic, Intracorporeal, Robotic left colectomy

## Abstract

This video vignette demonstrates a robotic sigmoid colectomy with iso-peristaltic intracorporeal colo-colonic anastomosis (ICA) utilising DaVinci Xi^®^ platform. The dissection employs an’inferior mesenteric vein first(IMV) approach’, a structured technique for training in robotic assisted left sided colon and rectal resections. Robotic ICA offers advantages over extracorporeal anastomosis, enabling the surgeon to remain at the console for the entire procedure, thus enhancing surgical flow. Based on the literature ICA technique potentially improves patient outcomes, with reported reduction in incisional hernia, surgical site infections, and postoperative pain, particularly when Pfannenstiel incision is used for extraction of the specimen compared to periumbilical incision. The robot precision facilitates suturing including hand-sewn anastomosis formation. Our video featured a case of 72-year-old male diagnosed with mid sigmoid cancer, highlighting the practical application of ‘IMV First' approach. The total operative time was 120 min with minimal blood loss. Patient was discharged on postoperative day 3 with no complications within 30 days. Postoperative histology-pT3N0M0R0.

**Supplementary Information:**

The online version contains supplementary material available at 10.1007/s10151-025-03249-6.

## Introduction

Robotic assisted surgery with its advantages like 3D visualization, tremor filtration, high dexterity and precision, has significantly advanced the safety and precision of colorectal cancer procedures over the years [1]. Intracorporeal anastomosis(ICA) for right-sided resections has gained popularity, offering benefits such as better cosmesis, reduced incisions and consequently less pain and quicker patient recovery [2].

Historically, left-sided resections involved extracorporeal anastomosis formation, a practice that partially continued with the introduction of robotic surgery. This often necessitates the surgeon to scrub for the anastomosis part of the procedure, frequently requiring partial or full undocking of the robot to exteriorize the specimen, perform the resection and place the anvil of the circular stapler. Such interruptions disrupt the surgical flow and prolong the procedure length. While some intracorporeal anastomosis techniques are described for left sided and rectal resections using natural orifice extraction methods this is technically challenging procedure [3]. Given that majority of these depend on lesion distance from anal verge and the available circular stapler type, we propose a simple and reproducible method using robotic linear stapler for high rectal and left sided colonic lesion.

## Aim

Given the limited existing literature describing side-to side intracorporeal anastomosis for left sided colorectal lesions, this video aims to showcase a simple and reproducible technique using robotic platform.

## Case

This was a case of a 72-year-old male with performance status of 0 and BMI of 29 kg/m^2^ without no prior past surgical history. He was evaluated by his general practitioner for change in bowel habits towards diarrhea and referred to secondary care for further investigations due to positive faecal immunochemical test. Colonoscopy revealed a stenotic, mid sigmoid colon moderately differentiated adenocarcinoma at 30 cm from anal verge clinically staged as T4a N0 M0. Following colorectal cancer multidisciplinary team discussion, he underwent a robotic sigmoid colectomy using DaVinci Xi® platform. The surgical approach involved medial-to-lateral dissection below inferior mesenteric vein (IMV). High ligation of IMV at the level of the pancreas and lateral mobilisation of sigmoid and descending colon performing partial splenic flexure mobilisation. Double window approach to inferior mesenteric artery (IMA) and low IMA control using Da Vinci Vessel Sealer Extend®. Mesocolon divided using energy device until proximal and distal resection margins are reached within the oncological limits. Distal and proximal colonic divisions were performed with a SureForm® 60 stapler. An iso-peristaltic colo-colonic anastomosis was created and the technical orifice closed with absorbable sutures in 2 layers. The total operative time was 120 min with minimal blood loss recorded 50 millilitres. Patient underwent an uneventful recovery and discharged home on day 3 without complications recorded within 30 days post-surgery. Postoperative histology confirmed pT3N0M0R0 moderately differentiated adenocarcinoma.

## Discussion

The transition toward intracorporeal anastomosis techniques in colorectal surgery represents a significant step in enhancing postoperative recovery. While the benefits of intracorporeal anastomosis are well-documented in right-sided resections, its application in left-sided procedures is increasingly recognised for its potential to further reduce surgical morbidity [4]. This video vignette illustrates the detailed steps of this method, aiming to demonstrate its feasibility, efficiency, and potential to minimise staple-line intersections, thereby optimising the surgical workflow and improving outcomes for patients undergoing left-sided colorectal resections.

The high dexterity of robotic instrumentation, coupled with superior 3D visualisation, allows for meticulous dissection and precise suturing within the confined space. These features are critical for ensuring a safe anastomosis and achieving negative resection margins.

One of the key advantages of this method is the elimination of the need for colonic extraction for anvil placement. In extracorporeal techniques, additional colonic mobilisation is often required to reach extraction site. This process carries risks, including mechanical stress such as unintentional twisting or mesenteric stretching, and vascular compromise that may lead to oedema or bleeding. In contrast, the ICA approach reduces tension on the tissues, thereby minimising the risk of injury and supporting faster postoperative functional recovery. By optimising the surgical workflow and minimising staple-line intersections, this technique aims to lower the incidence of complications and ultimately improve patient outcomes.

We believe the technique described is reproducible for other surgical teams familiar with robotic platforms. By standardising the steps of the anastomosis, surgeons can minimise the complexities of the left-sided resection while maximising the benefits of minimally invasive surgery.

While this vignette demonstrate feasibility and efficiency, we acknowledge that further randomised prospective studies are the necessary to be done. Such trials will be essential to prove the long-term clinical advantages of this intracorporeal technique over traditional extracorporeal methods.

## Supplementary Information

Below is the link to the electronic supplementary material.

Supplementary file1 (MP4 995312 KB)

## Data Availability

No datasets were generated or analysed during the current study.
